# Osteosarcoma Phenotype Is Inhibited by 3,4-Methylenedioxy-*β*-nitrostyrene

**DOI:** 10.1155/2012/479712

**Published:** 2012-05-31

**Authors:** Patrick J. Messerschmitt, Ashley N. Rettew, Nicholas O. Schroeder, Robert E. Brookover, Avanti P. Jakatdar, Patrick J. Getty, Edward M. Greenfield

**Affiliations:** ^1^Department of Orthopaedics, University Hospitals Case Medical Center, Case Western Reserve University, 11100 Euclid Avenue, Cleveland, OH 44106, USA; ^2^Department of Pathology, University Hospitals Case Medical Center, Case Western Reserve University, 11100 Euclid Avenue, Cleveland, OH 44106, USA; ^3^Case Comprehensive Cancer Center, University Hospitals Case Medical Center, Case Western Reserve University, 11100 Euclid Avenue, Cleveland, OH 44106, USA

## Abstract

*β*-nitrostyrene compounds, such as 3,4-methylenedioxy-*β*-nitrostyrene (MNS), inhibit growth and induce apoptosis in tumor cells, but no reports have investigated their role in osteosarcoma. In this study, human osteosarcoma cell families with cell lines of varying tumorigenic and metastatic potential were utilized. Scrape motility assays, colony formation assays, and colony survival assays were performed with osteosarcoma cell lines, both in the presence and absence of MNS. Effects of MNS on human osteoblasts and airway epithelial cells were assessed in monolayer cultures. MNS decreased metastatic cell line motility by 72–76% and colony formation by 95–100%. MNS consistently disrupted preformed colonies in a time-dependent and dose-dependent manner. MNS had similar effects on human osteoblasts but little effect on airway epithelial cells. An inactive analog of MNS had no detectable effects, demonstrating specificity. MNS decreases motility and colony formation of osteosarcoma cells and disrupts preformed cell colonies, while producing little effect on pulmonary epithelial cells.

## 1. Introduction

Osteosarcoma, the most common malignant bone tumor in children and adolescents, affects nearly 560 new individuals each year in the United States [1]. Among other prognostic indicators, metastases on presentation and poor response to preoperative chemotherapy are markers for a dismal outcome [2–4]. Approximately 20% of new cases present with clinically detectable metastases; however, current protocols provide treatment addressing presumed presence of pulmonary micrometastases [2, 5]. The pulmonary system is the most common site of distant metastasis with distant bony sites second [4]. The addition of liposomal muramyl tripeptide phosphatidylethanolamine to chemotherapeutic regimens and surgical resection has resulted in five-year survival rates of 78% and 53% for patients without and with metastases, respectively [6, 7]. The continued high mortality rates for children and adolescents with metastatic osteosarcoma outlines the need for further research into the development of novel therapeutics.

Beta-nitrostyrenes have recently been found to inhibit tumor cell growth and induce apoptosis through disruption of tubulin polymerization, inhibition of protein phosphatases, and inhibition of telomerase activity [8–10]. 3,4-methylenedioxy-*β*-nitrostyrene (MNS), a synthesized *β*-nitrostyrene, is reported to inhibit Syk and Src tyrosine kinase activity [11, 12]. Tyrosine kinases participate in the regulation of cell proliferation, migration, motility, colony formation, invasiveness, and apoptosis [13, 14]. Thus, tyrosine kinases participate in important processes which are critical for the survival, growth, and metastatic potential of tumor cells. Inhibitory molecules of receptor and non-receptor tyrosine kinases targeting epidermal growth factor receptor, nerve growth factor receptor, ErbB-2/*neu*, insulin-like growth factor-1 receptor, hepatocyte growth factor receptor (*met*/HGF-R), platelet-derived growth factor receptors, and Janus family protein kinases have been investigated as potential chemotherapeutic agents for the treatment of osteosarcoma, for their roles in reducing tumor cell growth, motility, invasiveness, and colony formation [14, 15]. Syk, a non-receptor tyrosine kinase, is involved in signaling pathways and cell-cycle control with the majority of research to this date focused on the role of aberrant Syk expression and/or signaling in hematopoietic lineage cells, hematopoietic cancer, lung carcinoma, and gastric tumors [16]. Recent evidence demonstrates the presence of activated Syk in human osteosarcoma cells [17, 18]. Syk-dependent signaling pathways target the phosphorylation and activation of phosphatidylinositol 3-kinase and phospholipase C-*γ*_2_ [19, 20]. Additionally, Syk signaling pathways interact with members of the non-receptor tyrosine kinase Src family [21, 22]. c-Src expression results in the activation of critical participants for cell growth, angiogenesis, migration and invasion in several types of sarcomas, including osteosarcoma [23–25].

First, we asked whether MNS, a synthesized *β*-nitrostyrene derivative, alters the motility, colony formation, and colony survival of osteosarcoma cell lines. Second, we asked whether MNS affects normal, human osteoblasts and small airway epithelial cells since metastases primarily involve lung parenchyma and nasal administration of chemotherapeutics is being developed for osteosarcoma [26]. 

## 2. Materials and Methods

Two families of genetically-related osteosarcoma cell lines were used, with each parental cell line harvested from human osteosarcoma tissue. The TE85 family was obtained from the American Type Culture Collection (Manassas, VA, USA) and included a parental cell line (TE85: little tumorigenic or metastatic potential) and two derivative cell lines (MNNG: tumorigenic but weakly metastatic; 143B: highly tumorigenic and metastatic) [27]. The SAOS-2 family was obtained from Dr. E. Kleinerman, MD (Anderson Cancer Center, Houston, TX, USA) and included a parental cell line (SAOS-2: little tumorigenic or metastatic potential) and a derivative cell line (LM7: highly tumorigenic and metastatic) [28, 29]. Unless otherwise specified, all cell cultures were maintained according to a previously described method [14]. Cells were harvested during the mid-log phase of growth for all experiments.

 The effects of small molecule inhibitors were used in tests of motility, nonadherent colony formation, or nonadherent colony survival. Small-molecule inhibitors and inactive analogues were obtained from Calbiochem (San Diego, CA, USA) and Sigma-Aldrich (St. Louis, MO, USA) and the concentrations of small molecule inhibitors used were based on publications demonstrating the effective concentrations in intact cell assays (Table 1). Inhibitors were dissolved in dimethyl sulfoxide (DMSO; Sigma, St. Louis, MO, USA), with the exception of Syk IV (H_2_O), and aliquots were stored at −20°C.

Scrape motility assays were performed similarly to a previously described method [14, 34, 35]. Briefly, cells (1.0 × 10^5^ per 9.6 cm^2^ well) were cultured to form a confluent monolayer before scrapes were made using a 1 mL pipette tip. The chambers were incubated in the presence of inhibitors or vehicle controls. Each scrape was photographed immediately and at the indicated time points using the 10x objective on a Leica DM IRB inverted, phase contrast microscope (Leica Microsystems, Deerfield, IL, USA). Scrape widths were measured with ImageJ (NIH). Motility was calculated by subtracting the scrape width at the indicated time points from the initial scrape width and dividing by two. 

Colony formation assays were performed similarly to a previously described method [14, 27]. Briefly, 8.0 × 10^3^ cells were suspended in rat tail type I collagen gel (BD Biosciences, Bedford, MA, USA) overlying a lower layer of collagen gel in a 2 cm^2^ culture well. Gels were then covered with media plus inhibitors, inactive analogue, or vehicle controls. Media, inhibitors, and vehicle controls were changed every 48 hours. Colonies (≥5 intact cells) were counted after 3 days (TE85, MNNG, 143B) or 6 days (SAOS-2, LM7) using phase contrast microscopy. 

Non-adherent colony survival assays were performed by a modification of the colony formation assays. Briefly, colonies were allowed to form as described in the previous paragraph for 3 (TE85, MNNG, 143B) or 6 (SAOS-2, LM7) days and then gels were incubated with 1 mL of media containing 10% FBS plus sufficient levels of the small molecule inhibitors, inactive analogue, or vehicle controls (DMSO or H_2_O depending on the inhibitor) to obtain the indicated concentrations. Media, inhibitors, and vehicle controls continued to be changed every 48 hours. Colonies (≥5 intact cells) were analyzed after the indicated times using phase contrast microscopy. 

Membrane integrity was assessed by DAPI staining of non-permeabilized cells. DAPI was added to the media overlying collagen gels 8 hours after initial treatment with MNS, inactive analogue, or vehicle control. Phase contrast and fluorescent microscopy images were obtained at the 24 hour time point.

The effects of MNS on normal, human osteoblasts (Cambrex, East Rutherford, NJ) and normal, human, small airway epithelial cells (ATCC, Washington, D.C., USA) were assessed in monolayer cultures. Briefly, cells (1.0 × 10^5^ per 9.6 cm^2^ well) were cultured to form a confluent monolayer. Small airway epithelial cells were cultured in Airway epithelial cell basal medium (ATCC, Washington, D.C., USA) supplemented with bronchial epithelial cell growth kit (ATCC, Washington, D.C., USA). Cells were incubated in the presence of inhibitor, inactive analogue, or vehicle control. At the indicated time points, media was changed to remove non-adherent cells and phase contrast microscopy was used to analyze the cells.

 Expression of src and syk mRNAs was determined by real time RT-PCR as described previously [36]. Total RNA was isolated using the ToTALLY RNA kit (Ambion, Austin, TX, USA). RNA (0.2 *μ*g) was reverse transcribed into cDNA using SuperScript II reverse transcriptase (Invitroge, Carlsbad, CA, USA). Quantitative RT-PCR was performed with SYBRgreen PCR Master Mix (BioRad, Hercules, CA, USA) and the 7500 real-time PCR systems and Sequence Detection Software (Applied Biosystems, Foster City, Ca, USA). Gene expression was analyzed using a standard curve as previously described [36].

The effects of MNS on activity of tyrosine kinases was determined biochemically using recombinant forms of the kinases (KinaseProfiler Service, Millipore, Billerica, MA) as described [37].

Statistical analysis was determined by ANOVA with the Bonferroni post-hoc tests performed for all analyses (SigmaStat, San Jose, CA, USA). All figures illustrate mean ± standard error of the mean. 

## 3. Results

**Figure 1:**
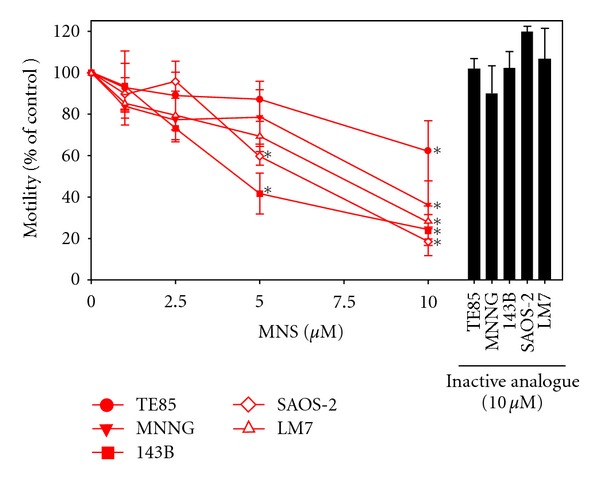
MNS reduced motility in a dose-dependent manner in the osteosarcoma cell lines. Motility was measured 4 hours after scraping in the presence of the indicated concentrations of MNS, 10 *μ*M of the inactive analogue, or 1% DMSO as a vehicle control. Data are represented as means ± SE of three independent experiments, each with four scrapes per group. Average migration distance for vehicle control groups were 58 *μ*m for TE85 cells, 41 *μ*m for MNNG cells, 60 *μ*m for 143B cells, 26 *μ*m for SAOS-2 cells, and 18 *μ*m for LM7 cells. Asterisks denote differences in motility compared with the vehicle control group (10 *μ*M MNS: TE85 (*P* = 0.020), MNNG (*P* = 0.009), 143B (*P* < 0.001), SAOS-2 (*P* < 0.001), LM7 (*P* = 0.001); 5 *μ*M MNS: 143B (*P* < 0.001), SAOS-2 (*P* = 0.002)).

MNS slowed motility of all osteosarcoma cell lines in a dose-dependent manner (Figure 1). The motility of the metastatic 143B cell line was decreased by 52% at 5 *μ*M of MNS and 76% at 10 *μ*M of MNS, while the motility of the tumorigenic but non-metastatic MNNG cells was decreased by 22% at 5 *μ*M of MNS and 64% at 10 *μ*M of MNS (Figure 1). MNS had less of an effect on the non-tumorigenic, parental TE85 cells reducing the motility by 13% at 5 *μ*M of MNS and 38% at 10 *μ*M of MNS (Figure 1). MNS reduced motility of the non-tumorigenic, parental SAOS-2 and metastatic LM7 cell lines by 40% and 31% at 5 *μ*M of MNS, respectively, and by 81% and 72% at 10 *μ*M of MNS, respectively (Figure 1). The inactive analogue of MNS did not alter the motility of the osteosarcoma cell lines (Figure 1). 

**Figure 2:**
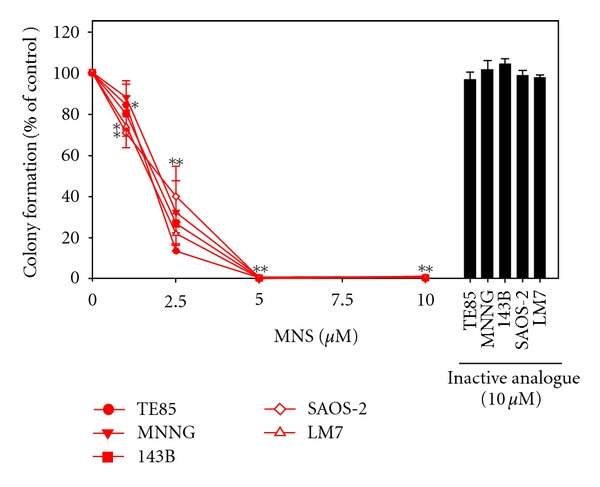
MNS reduced colony formation in a dose-dependent manner in the osteosarcoma cell lines. Colonies were counted 24 hours after plating in collagen gel in the presence of MNS, an inactive analogue, or 1% DMSO as a vehicle control. Data are represented as means ± SE of three independent experiments, each with three wells per group. Average colony formation per well for vehicle control groups were 141 for TE85 cells, 133 for MNNG cells, 187 for 143B cells, 152 for SAOS-2 cells, and 115 for LM7 cells. Asterisks denote *P* < 0.05 compared with the vehicle control group. Double asterisks indicate all cell lines were *P* < 0.05 at 2.5, 5, and 10 *μ*M MNS (10 *μ*M MNS: TE85 (*P* < 0.001), MNNG (*P* < 0.001), 143B (*P* < 0.001), SAOS-2 (*P* < 0.001), LM7 (*P* < 0.001); 5 *μ*M MNS: TE85 (*P* < 0.001), MNNG (*P* < 0.001), 143B (*P* < 0.001), SAOS-2 (*P* < 0.001), LM7 (*P* < 0.001); 2.5 *μ*M MNS: TE85 (*P* < 0.001), MNNG (*P* < 0.001), 143B (*P* < 0.001), SAOS-2 (*P* < 0.001), LM7 (*P* < 0.001); 1 *μ*M MNS: TE85 (*P* = 0.002), SAOS-2 (*P* = 0.040), LM7 (*P* = 0.0340).

MNS reduced the non-adherent colony formation of all osteosarcoma cell lines in a dose-dependent manner (Figure 2). Colony formation in collagen gels was reduced by 95–100% for all osteosarcoma cells lines in the presence of 5.0 *μ*M MNS (Figure 2). The inactive analogue of MNS did not alter colony formation by the osteosarcoma cell lines (Figure 2). 

**Figure 3:**
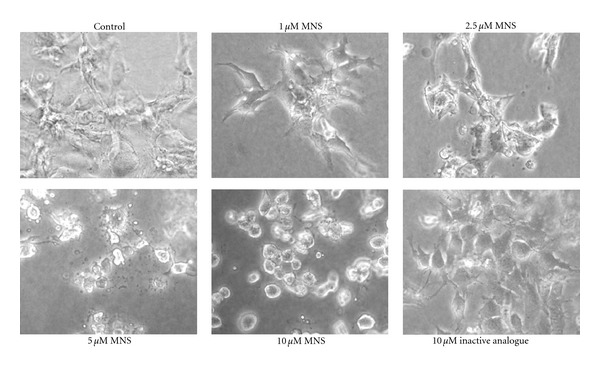
MNS disrupts preformed metastatic 143B cell colonies in a dose-dependent manner. 143B cell colonies 4 hours after incubation in the presence of increasing concentrations of MNS (1, 2.5, 5, and 10 *μ*M), 10 *μ*M of the inactive analogue, or 1% DMSO as a vehicle control. This figure shows representative images from three independent experiments, each with three wells per group.

MNS rapidly and dose-dependently disrupted preformed colonies of 143B cells (Figure 3). No obvious effects were seen at 1 *μ*M of MNS (Figure 3). With MNS concentrations of 2.5 *μ*M and 5 *μ*M, increasing cellular fragmentation is demonstrated and membrane projections are still visible (Figure 3). At 10 *μ*M of MNS, cells lose their membrane projections, become more “rounded” morphologically, and lose their cohesiveness as a colony (Figure 3). These detrimental changes occurred within 4 hours of exposure (Figure 3).

**Figure 4:**
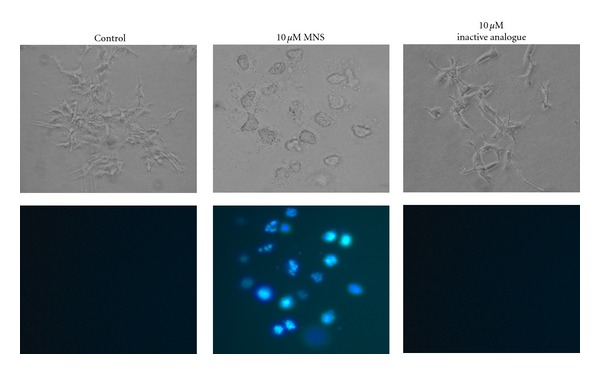
MNS disrupts preformed 143B colonies. Images of the metastatic 143B cell colonies incubated for 24 hours in presence of 10 *μ*M MNS, 10 *μ*M of the inactive analogue, or 1% DMSO as a vehicle control. The membrane-impermanent fluorescent stain 4′, 6-diamidino-2-phenylindole (DAPI, 0.1 *μ*g/mL) was added 8 hours after incubation began. Fluorescent microscopy was used to obtain photomicrographs (bottom panel). This figure shows representative images from three independent experiments, each with two to three wells per group.

Cell death was confirmed based on loss of membrane integrity, which occurs within 24 hours of exposure as assessed by DAPI staining (Figure 4).

**Figure 5:**
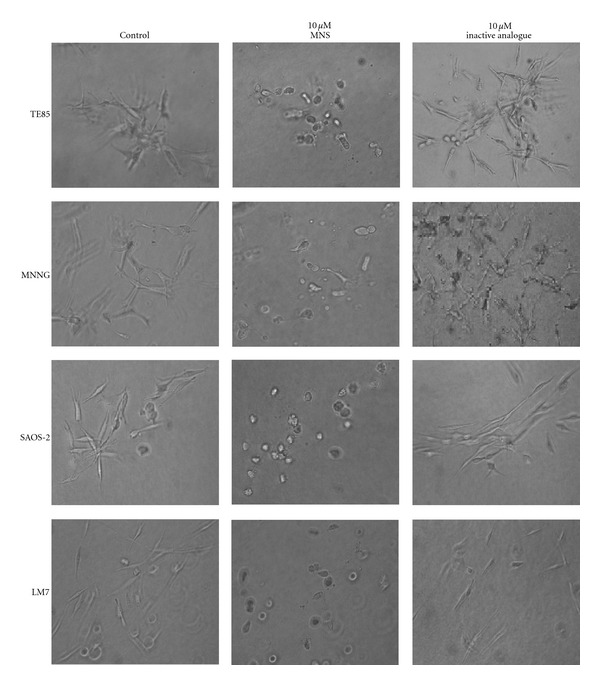
MNS disrupts preformed colonies of the osteosarcoma cell lines. Representative photomicrographs for each cell line 24 hours after incubation in the presence of 10 *μ*M MNS, 10 *μ*M of the inactive analogue, or 1% DMSO as a vehicle control. Each experiment was performed in triplicate, each with three wells per group.

TE85, MNNG, SAOS-2, and LM7 osteosarcoma cell lines were also disrupted in a similar dose-dependent manner (only 10 *μ*M dose shown, Figure 5). The inactive analogue of MNS had no detectable effect on the osteosarcoma cells (Figures 3, 4, 5).

**Figure 6:**
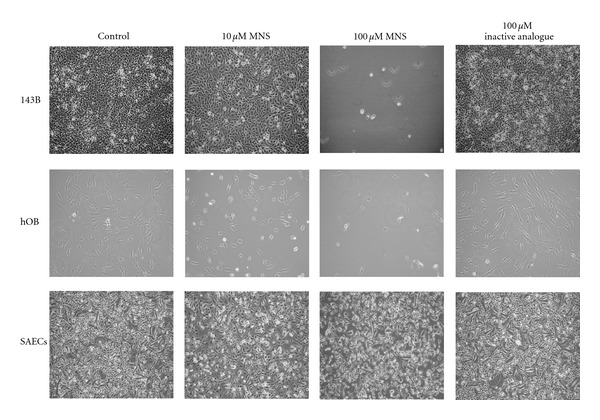
MNS also disrupts monolayers of osteosarcoma cells and osteoblasts but does not alter monolayers of small airway epithelial cells. 143B metastatic osteosarcoma cell line, normal human osteoblasts (hOB), and normal human small airway epithelial cells (SAEC) after culture for 24 hours in the presence of 10 *μ*M MNS, 100 *μ*M MNS, 100 *μ*M of the inactive analogue, or 1% DMSO as a vehicle control. Figure shows representative images from three independent experiments, each with three wells per group.

Monolayer cultures were used to compare the effects of MNS on the osteosarcoma cells with its effects on primary cultures of normal, human osteoblasts and normal, human small airway epithelial cells since neither of the normal cell types form non-adherent colonies. Moreover, mature osteoblasts and epithelial cells are both organized in monolayers in normal, physiologic conditions. In monolayer culture, 10 *μ*M MNS had no detectable effect on any of the cell types but 100 *μ*M MNS substantially disrupted both the 143B osteosarcoma cells and the normal, human osteoblasts (Figure 6). On the contrary, 100 *μ*M MNS only induced a minimal morphological change in the small airway epithelial cells, but these pulmonary cells remained alive and viable (Figure 6). Thus, the effects seen here by MNS preferentially disrupting metastatic osteosarcoma cells while sparing pulmonary cells could be utilized in chemotherapeutic drug development. Osteosarcoma patients with pulmonary metastases may benefit from an intranasally-based MNS compound and experience less drug-related morbidity.

**Figure 7:**
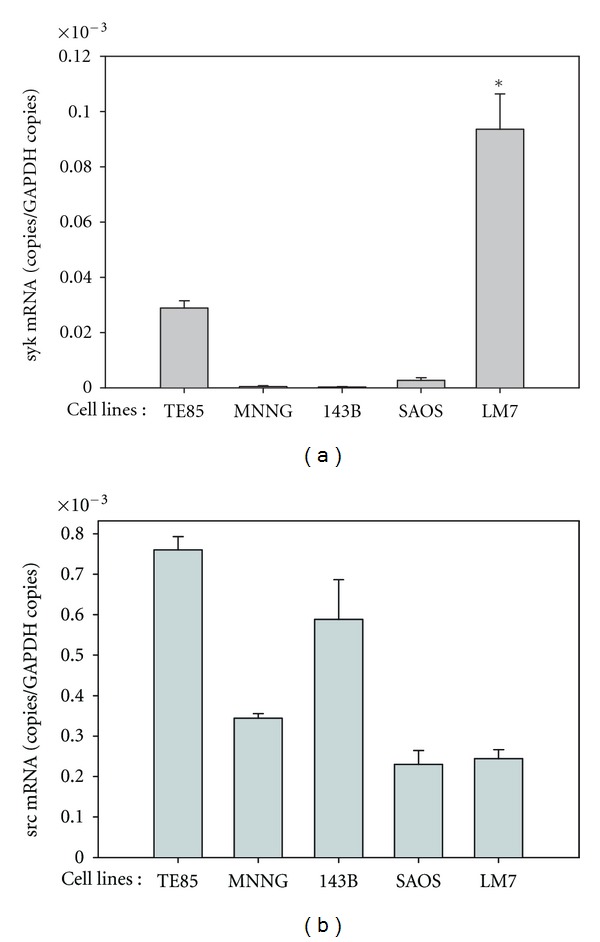
Expression of syk (a) and src (b) mRNA levels in the osteosarcoma cell lines at mid-log phase. mRNA levels were normalized to those of GAPDH. The asterisk denotes *P* < 0.001 compared with the mRNA levels of the non-metastatic SAOS-2 cell line. Each reaction was performed in triplicate.

MNS has been reported to inhibit the tyrosine kinase activity of src and syk [11, 12]. Expression of mRNAs encoding these kinases was therefore measured in the osteosarcoma cell lines by real-time RT-PCR. Syk was reliably detected only in the TE85 and LM7 cell lines (Figure 7(a)), while src was expressed at low, but reproducible levels in all five cell lines (Figure 7(b)). 

**Figure 8:**
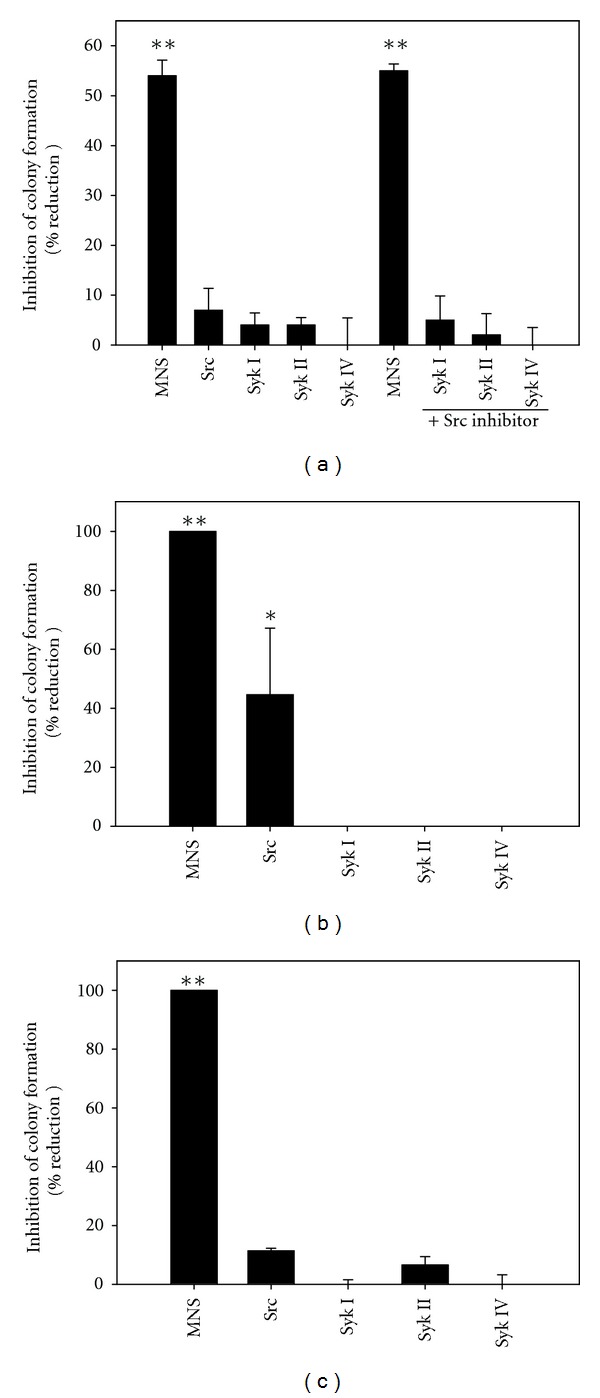
Specific tyrosine kinase inhibitors of syk and src do not reduce motility or colony formation by the metastatic 143B cells (panel (a) & (b)) or colony formation by the non-tumorigenic/non-metastatic TE85 cells (panel (c)). Motility and colony formation assays were performed in the presence of tyrosine kinase inhibitors or 1% DMSO as a vehicle control. Bars represent the means ± standard error of the mean of three individual experiments (scrape motility assays with four to six scrapes per group; colony formation assays with three wells per group). Double asterisks denote *P* < 0.001 compared with the vehicle control groups; single asterisk (panel (b)) denotes *P* = 0.012.

The effect of MNS on the osteosarcoma cell lines was compared with a src inhibitor and three additional syk inhibitors [12, 31, 32]. Although the effect of MNS was confirmed in these experiments (Figures 8(a)–8(c)), neither the src or other syk inhibitors, alone or in combination, mimicked the effects of MNS on motility (Figure 8(a)) or colony formation (Figures 8(b) and 8(c)). These results suggest that the effects of MNS are due either to the combined inhibition of Src or Syk and an additional tyrosine kinase(s) or to inhibition of tyrosine kinases other than Src and Syk.

**Figure 9:**
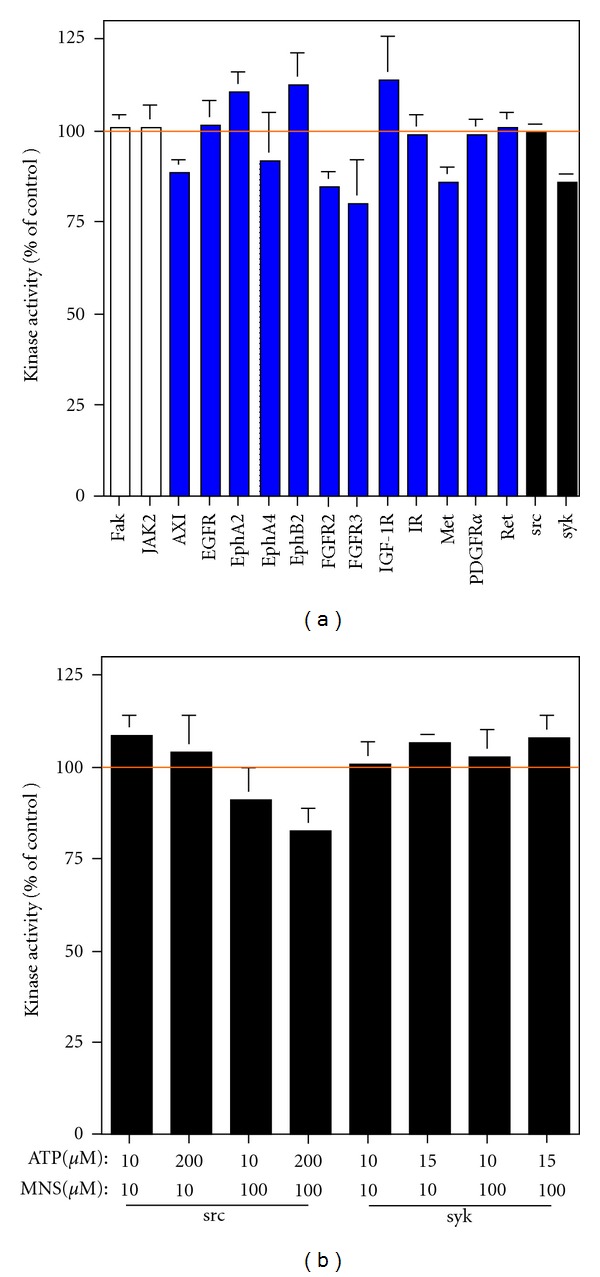
MNS does not inhibit activity of any of the 16 tyrosine kinases that were tested, including syk and src. In panel (a), the effect of 10 *μ*M MNS was examined in the presence of the concentration of ATP that approximates the Km for the individual kinase. In panel (b), the effect of 10 or 100 *μ*M MNS was examined in the presence of 10 *μ*M ATP or the concentration of ATP that approximates the Km for src or syk. All assays were performed in either triplicate or quadruplicate.

To determine what other tyrosine kinases might be inhibited by MNS, its effects on the activity of recombinant kinases was determined. As expected [11, 12], MNS did not effect activity of Fak or JAK2 (Figure 9(a)). Figure 9(a) also shows that MNS had no effect on activity of the twelve tyrosine kinases that we have found to be activated in the 143B and LM7 cells [38]. In contrast to the results by Wang et al. [11, 12], MNS also did not inhibit activity of Src or Syk, even when the MNS concentration was increased to 100 *μ*M and/or the ATP concentration in the reaction was reduced to 10 *μ*M (Figures 9(a) and 9(b)).

## 4. Discussion

MNS is an attractive lead compound for the development of a novel chemotherapeutic option for patients suffering from osteosarcoma. MNS significantly reduced osteosarcoma cell motility and colony formation, but the effects were more substantial in colony formation assays. MNS consistently disrupted preformed colonies in a time-dependent and dose-dependent manner while having little effect on airway epithelial cells. In contrast, the inactive analogue of MNS did not cause deleterious effects for either osteosarcoma or airway epithelial cells, and thus, demonstrated specificity of the MNS effects. 

Other authors have reported that MNS inhibits the tyrosine kinase activity of syk, and at higher concentrations, will inhibit the kinase activity of src [11, 12]. However, the concentration of MNS used in this study was below the reported IC_50_ (29.3 *μ*M) for inhibition of src and therefore likely does not account for the observed results [12]. Additionally, the use of alternative syk inhibitors did not produce reduction in motility, colony formation, or colony survival as seen in the presence of MNS. The combination of the src inhibitor with either the Syk I, Syk II, or Syk IV inhibitors also did not produce results similar to MNS alone. Furthermore, biochemical assays showed that MNS did not inhibit the activity of syk, src, or the 12 other tyrosine kinases that have been shown to be activated in these cell lines [38]. Our results demonstrate the effect of MNS in the tumorigenic and metastatic assays is not due to syk or src inhibition, but rather to another mechanism(s) which is yet to be identified. The uncertainty as to the molecular target of MNS does not preclude its usefulness as a lead compound for development of novel chemotherapeutics.

Potential mechanisms for explanation of the observed results are based on the core structure of MNS. MNS is derived from a *β*-nitrostyrene moiety with an aromatic ring and nitrovinyl side chain, both of which are critical to the drug's biological activity [39]. Compounds with a similar structure have been implicated in growth inhibition and pro-apoptotic functions [8–10, 39–41]. *β*-nitrostyrene derivatives exihibit antiproliferative properties by disrupting tubulin polymerization, which leads to conformational changes in tubulin, and interrupts cellular mitosis [10, 40]. The pro-apoptotic effects of *β*-nitrostyrene compounds is due to their inhibition of cellular protein phosphatases, including serine/threonine phosphatases 1 and 2A (PP1 and PP2A) and PTB1 [8, 42, 43]. For example, trans-*β*-nitrostyrene has been shown to inhibit protein phosphatases PP2A and PTB1 leading to apoptosis in colon cancer cells [43]. Inhibition of these protein phosphatases disrupts key cellular signal transduction, and in the case of PP2A inhibition, Bcl-2 is consequently hyperphosphorylated, thus inhibiting Bcl-2 anti-apoptotic activity [43, 44]. In another study, McNamara et al. demonstrated nitrostyrene induction of chromatin condensation, caspase activation, and membrane blebbing leading to apoptosis in a Burkitt's lymphoma derived cell line [41]. Furthermore, Werner et al. reported rapid onset of action as 50% decrease in colon cancer cell viability was demonstrated after just 8 hours of exposure to the **β**-nitrostyrene derivative [43].

*beta*-Nitrostyrene derivatives have also been shown to be capable of telomerase inhibition [9]. One adaptive and protective characteristic of various tumor cells is production of telomerase which prevents telomere erosion during repeated cell cycles [9, 45]. Telomerase effectively prevents cell senescence and prolongs the life of tumor cells [9, 45]. Cervical cancer cells treated with **β**-nitrostyrene compounds are shown to possess telomere shortening and cellular senescence in a dose-dependent manner [9].

 The current literature lacks data involving safety profiles for *β*-nitrostyrene derivatives regarding side effects following human administration. Compounds which possess target specificity are ideal as they generally result in fewer side effects and better patient compliance. Since MNS and other B-nitrosytrenes kill some but not all cancer cells [41], it is likely that the effects are specific for certain cell types. In this study, the presence of MNS resulted in cell disruption and death, both in the osteosarcoma cells and normal, human osteoblasts. However, MNS application did not disrupt or kill small airway epithelial cells, but instead, the pulmonary cells remained viable. These results outline the interest in MNS as new chemotherapeutic option, potentially with a safety profile to permit nasal administration. 

To the best of the author's knowledge, this is the first report of utilizing the colony survival assays as outlined within this paper. The concept of allowing tumorigenic colony formation within a collagen matrix prior to the addition of inhibitor may mimic treatment of patients with pre-existing metastasis. The concept is important as all osteosarcoma patients are presumed to have micrometastases at time of diagnosis, approximately 20% of patients actually present with clinically detectable metastases, and the leading cause of death is pulmonary failure secondary to metastatic disease [4]. Future goals include validating the proposed clinical correlation of nonadherent colony survival assays in an osteosarcoma animal model possessing metastatic pulmonary nodes. The colony survival assay would likely be helpful in the development of other chemotherapeutic compounds.

The *in vitro* nature of the motility, colony formation, and colony survival experiments is a limitation of the study. However, the *in vitro* motility and colony formation of the human osteosarcoma cell lines correlates to their *in vivo* tumorigenic and metastatic potential [14, 27, 29]. Future studies are needed to determine whether the results of MNS are replicated *in vivo*.

The decrease in motility, colony formation, and colony survival of osteosarcoma cells following *in vitro* treatment with MNS are encouraging. Future studies to identify the target(s) responsible for the effects of MNS on osteosarcoma cells would offer the possibility of developing chemotherapeutics that are even more specific for the target(s).

## 5. Conclusion

 MNS decreases the motility and colony formation of osteosarcoma cells. MNS disrupts preformed osteosarcoma cell colonies while producing little effect on pulmonary epithelial cells. Further investigations will unveil the full potential of MNS as a new and useful chemotherapeutic drug to be used clinically as part of a multi-drug strategy for patients suffering from osteosarcoma.

## Figures and Tables

**Table 1:** Small molecule inhibitors and inactive MNS analogue.

Inhibitor	Catalog number	Concentration used	Reference number
MNS (Syk III)	574713^a^	20 *μ*M (5x IC_50_)	[11, 12]
Src Inhibitor (SU6656)	572635^a^	0.5 *μ*M (2x IC_50_)	[30]
Syk I	574711^a^	1.5 *μ*M (5x IC_50_)	[31]
Syk II	574712^a^	2.3 *μ*M (5x IC_50_)	[32]
Syk IV (Bay 61-3606)	574714^a^	0.2 *μ*M (5x IC_50_)	[33]
Inactive MNS Analogue (MCA)	146242^b^	10 *μ*M (IC_50_ > 100 *μ*M)	[11]

^a^: Calbiochem; ^b^: Sigma-Aldrich.

## References

[1] Surveillance E, End Results (SEER) Program (2008). *SEER Stat Database: Incidence - SEER 17 Regs Limited-Use + Hurricane Katrina Impacted Louisiana Cases, Nov 2007 Sub (1973–2005 varying)—Linked to County Attributes—Total U.S., 1969–2005*.

[2] Bielack S, Kempf-Bielack B, Delling G (2002). Prognostic factors in high-grade osteosarcoma of the extremities or trunk: an analysis of 1,702 patients treated on neoadjuvant cooperative osteosarcoma study group protocols. *Journal of Clinical Oncology*.

[3] Davis AM, Bell RS, Goodwin PJ (1994). Prognostic factors in osteosarcoma: a critical review. *Journal of Clinical Oncology*.

[4] Messerschmitt PJ, Garcia RM, Abdul-Karim FW, Greenfield EM, Getty PJ (2009). Osteosarcoma. *Journal of the American Academy of Orthopaedic Surgeons*.

[5] Meyers PA, Schwartz CL, Krailo M (2005). Osteosarcoma: a randomized, prospective trial of the addition of ifosfamide and/or muramyl tripeptide to cisplatin, doxorubicin, and high-dose methotrexate. *Journal of Clinical Oncology*.

[6] Chou AJ, Kleinerman ES, Krailo MD (2009). Addition of muramyl tripeptide to chemotherapy for patients with newly diagnosed metastatic osteosarcoma: a report from the Children’s Oncology Group. *Cancer*.

[7] Meyers PA, Schwartz CL, Krailo MD (2008). Osteosarcoma: the addition of muramyl tripeptide to chemotherapy improves overall survival—a report from the children’s oncology group. *Journal of Clinical Oncology*.

[8] Kaap S, Quentin I, Tamiru D, Shaheen M, Eger K, Steinfelder HJ (2003). Structure activity analysis of the pro-apoptotic, antitumor effect of nitrostyrene adducts and related compounds. *Biochemical Pharmacology*.

[9] Kim JH, Kim JH, Lee GE, Lee JE, Chung IK (2003). Potent inhibition of human telomerase by nitrostyrene derivatives. *Molecular Pharmacology*.

[10] Mohan R, Rastogi N, Namboothiri INN, Mobin SM, Panda D (2006). Synthesis and evaluation of *α*-hydroxymethylated conjugated nitroalkenes for their anticancer activity: inhibition of cell proliferation by targeting microtubules. *Bioorganic & Medicinal Chemistry*.

[11] Wang WY, Hsieh PW, Wu YC, Wu CC (2007). Synthesis and pharmacological evaluation of novel *β*-nitrostyrene derivatives as tyrosine kinase inhibitors with potent antiplatelet activity. *Biochemical Pharmacology*.

[12] Wang WY, Wu YC, Wu CC (2006). Prevention of platelet glycoprotein IIb/IIIa activation by 3,4-methylenedioxy-*β*-nitrostyrene, a novel tyrosine kinase inhibitor. *Molecular Pharmacology*.

[13] Levitzki A, Mishani E (2006). Tyrphostins and other tyrosine kinase inhibitors. *Annual Review of Biochemistry*.

[14] Messerschmitt PJ, Rettew AN, Brookover RE, Garcia RM, Getty PJ, Greenfield EM (2008). Specific tyrosine kinase inhibitors regulate human osteosarcoma cells in vitro. *Clinical Orthopaedics and Related Research*.

[15] Hughes DPM, Thomas DG, Giordano TJ, McDonagh KT, Baker LH (2006). Essential erbB family phosphorylation in osteosarcoma as a target for CI-1033 inhibition. *Pediatric Blood and Cancer*.

[16] Coopman PJ, Mueller SC (2006). The Syk tyrosine kinase: a new negative regulator in tumor growth and progression. *Cancer Letters*.

[17] Rezzonico R, Schmid-Alliana A, Romey G (2002). Prostaglandin E2 induces interaction between hSlo potassium channel and Syk tyrosine kinase in osteosarcoma cells. *Journal of Bone and Mineral Research*.

[18] Yanagi S, Inatome R, Takano T, Yamamura H (2001). Syk expression and novel function in a wide variety of tissues. *Biochemical and Biophysical Research Communications*.

[19] Craxton A, Jiang A, Kurosaki T, Clark EA (1999). Syk and Bruton’s tyrosine kinase are required for B cell antigen receptor-mediated activation of the kinase Akt. *Journal of Biological Chemistry*.

[20] Stewart ZA, Pietenpol JA (2001). Syk: a new player in the field of breast cancer. *Breast Cancer Research*.

[21] Bradshaw JM (2010). The Src, Syk, and Tec family kinases: distinct types of molecular switches. *Cellular Signalling*.

[22] Turner M, Schweighoffer E, Colucci F, Di Santo JP, Tybulewicz VL (2000). Tyrosine kinase SYK: essential functions for immunoreceptor signalling. *Immunology Today*.

[23] Alvarez RH, Kantarjian HM, Cortes JE (2006). The role of Src in solid and hematologic malignancies: development of new-generation src inhibitors. *Cancer*.

[24] Hingorani P, Zhang W, Gorlick R, Kolb EA (2009). Inhibition of Src phosphorylation alters metastatic potential of osteosarcoma in vitro but not in vivo. *Clinical Cancer Research*.

[25] Shor AC, Keschman EA, Lee FY (2007). Dasatinib inhibits migration and invasion in diverse human sarcoma cell lines and induces apoptosis in bone sarcoma cells dependent on Src kinase for survival. *Cancer Research*.

[26] Worth LL, Jia SF, Zhou Z, Chen L, Kleinerman ES (2000). Intranasal therapy with an adenoviral vector containing the murine interleukin-12 gene eradicates osteosarcoma lung metastases. *Clinical Cancer Research*.

[27] Luu HH, Kang Q, Jong KP (2005). An orthotopic model of human osteosarcoma growth and spontaneous pulmonary metastasis. *Clinical and Experimental Metastasis*.

[28] Duan X, Jia SF, Zhou Z, Langley RR, Bolontrade MF, Kleinerman ES (2005). Association of *α*v*β*3 integrin expression with the metastatic potential and migratory and chemotactic ability of human osteosarcoma cells. *Clinical and Experimental Metastasis*.

[29] Jia SF, Worth LL, Kleinerman ES (1999). A nude mouse model of human osteosarcoma lung metastases for evaluating new therapeutic strategies. *Clinical and Experimental Metastasis*.

[30] Blake RA, Broome MA, Liu X (2000). SU6656, a selective Src family kinase inhibitor, used to probe growth factor signaling. *Molecular and Cellular Biology*.

[31] Lai JYQ, Cox PJ, Patel R (2003). Potent small molecule inhibitors of spleen tyrosine kinase (Syk). *Bioorganic & Medicinal Chemistry Letters*.

[32] Hisamichi H, Naito R, Toyoshima A (2005). Synthetic studies on novel Syk inhibitors. Part 1: synthesis and structure-activity relationships of pyrimidine-5-carboxamide derivatives. *Bioorganic & Medicinal Chemistry*.

[33] Yamamoto N, Takeshita K, Shichijo M (2003). The orally available spleen tyrosine kinase inhibitor 2-[7-(3,4-dimethoxyphenyl)-imidazo[1,2-c]pyrimidin-5-ylamino]-nicotinamide dihydrochloride (BAY 61-3606) blocks antigen-induced airway inflammation in rodents. *Journal of Pharmacology and Experimental Therapeutics*.

[34] Betapudi V, Licate LS, Egelhoff TT (2006). Distinct roles of nonmuscle myosin II isoforms in the regulation of MDA-MB-231 breast cancer cell spreading and migration. *Cancer Research*.

[35] Edin ML, Howe AK, Juliano RL (2001). Inhibition of PKA blocks fibroblast migration in response to growth factors. *Experimental Cell Research*.

[36] Dai JC, He P, Chen X, Greenfield EM (2006). TNF*α* and PTH utilize distinct mechanisms to induce IL-6 and RANKL expression with markedly different kinetics. *Bone*.

[37] Bain J, Plater L, Elliott M (2007). The selectivity of protein kinase inhibitors: a further update. *Biochemical Journal*.

[38] Rettew AN, Getty PJ, Greenfield EM Novel receptor tyrosine kinases in osteosarcoma identified by phosphoproteomic and functional genomic screening.

[39] Milhazes N, Calheiros R, Marques MPM (2006). *β*-Nitrostyrene derivatives as potential antibacterial agents: a structure-property-activity relationship study. *Bioorganic & Medicinal Chemistry*.

[40] Pettit RK, Pettit GR, Hamel E (2009). E-Combretastatin and E-resveratrol structural modifications: antimicrobial and cancer cell growth inhibitory *β*-E-nitrostyrenes. *Bioorganic & Medicinal Chemistry*.

[41] McNamara YM, Cloonan SM, Knox AJS (2011). Synthesis and serotonin transporter activity of 1,3-bis(aryl)-2-nitro-1-propenes as a new class of anticancer agents. *Bioorganic & Medicinal Chemistry*.

[42] Walsh AH, Cheng A, Honkanen RE (1997). Fostriecin, an antitumor antibiotic with inhibitory activity against serine/threonine protein phosphatases types 1 (PP1) and 2A (PP2A), is highly selective for PP2A. *FEBS Letters*.

[43] Werner JM, Eger K, Jürgen Steinfelder H (2007). Comparison of the rapid pro-apoptotic effect of trans-*β*-nitrostyrenes with delayed apoptosis induced by the standard agent 5-fluorouracil in colon cancer cells. *Apoptosis*.

[44] Deng X, Ito T, Carr B, Mumby M, May WS (1998). Reversible phosphorylation of Bcl2 following interleukin 3 or bryostatin 1 is mediated by direct interaction with protein phosphatase 2A. *Journal of Biological Chemistry*.

[45] Kim NW, Piatyszek MA, Prowse KR (1994). Specific association of human telomerase activity with immortal cells and cancer. *Science*.

